# Perceptions of quality of care in Midwife-led Birth Centres (MLBCs) in Uganda: Why do women choose MLBCs over other options?

**DOI:** 10.1016/j.wombi.2024.101612

**Published:** 2024-07

**Authors:** Rose Chalo Nabirye, Scovia Nalugo Mbalinda, Joshua Epuitai, Faith Nawagi, Sarah Namyalo, Andrea Nove, Oliva Bazirete, Kirsty Hughes, Sofia Castro Lopes, Sabera Turkmani, Mandy Forrester, Caroline S.E. Homer

**Affiliations:** a Busitema University, Tororo, Uganda; b Makerere University, Kampala, Uganda; c Uganda Private Midwives Association, Kampala, Uganda; d Novametrics Ltd, Duffield, United Kingdom; e University of Rwanda, Kigali, Rwanda; f Independent Consultant, Cape Town, South Africa; g Faculty of Health, University of Technology Sydney, Sydney, Australia; h Burnet Institute, Melbourne, Australia; i International Confederation of Midwives, The Hague, Netherlands

**Keywords:** Midwife-led care, Midwife-led Birthing Centres, Midwife, Uganda, Midwifery model of care, Childbirth care

## Abstract

**Background:**

Midwife-led birth centres (MLBCs) are associated with reduced childbirth interventions, higher satisfaction rates, and improved birth outcomes. The evidence on quality of care in MLBCs from low and middle-income countries (LMIC) is limited.

**Aim:**

This study aimed to explore the perceptions of women and midwives regarding the quality of care in four MLBCs in Uganda.

**Methods:**

A qualitative study was conducted in four MLBCs in Uganda. We conducted interviews with women and midwives in the MLBCs to explore their perceptions and experiences related to care in the MLBCs. The study obtained ethical approval. Deductive thematic analysis was used for data analysis.

**Results:**

Three key themes were identified regarding the perceptions of women and midwives about the quality of care in the MLBCs: providing respectful, and dignified care; a focus on woman-centred care; and reasons for choosing care in the MLBC. Women valued the respectful and humane care characterised by dignified and non-discriminatory care, non-abandonment, privacy, and consented care. The woman-centred care in the MLBC involved individualised holistic care, providing autonomy and empowerment, continuity of care, promoting positive birth experience, confidence in the woman’s own abilities, and responsive providers. Women chose MLBCs because the services were perceived to be available, accessible, affordable, with comprehensive and effective referral mechanisms.

**Conclusion:**

Women perceived care to be respectful, woman-centred, and of good quality. Global attention should be directed to scaling up the establishment of MLBCs, especially in LMIC, to improve the positive childbirth experience and increase access to care.

**Statement of significance**

**Problem**

Limited evidence exists in low-and middle-income countries regarding the reasons why women choose to give birth in MLBCs over other options.

**What is already known**

Women in other settings may choose MLBCs because of the need to avoid overmedicalisation of childbirth and need for positive childbirth experience.

**What this paper adds**

The study reflected findings of what women prioritised in MLBC from a resource constrained setting. Women valued the respectful, women-centred, and quality of care offered in the MLBCs settings.

## Introduction

Globally, there are 287,000 maternal deaths, 2.3 million neonatal deaths and 2 million stillbirths per year, most of which occur in low- and middle-income countries [1], [2]. However, ensuring universal coverage of midwife-delivered interventions could avert two-thirds of maternal and neonatal deaths and stillbirths [3]. These interventions could be delivered in the midwife-led continuity of care model, which is recommended by the World Health Organization in settings with a well-functioning midwifery programme [4]. Midwife-led care represents autonomous midwifery care in which the midwife is the lead healthcare provider for women having a low-risk pregnancy [5], [6]. Midwife-led care is associated with positive birth outcomes including a high likelihood of spontaneous vaginal birth, reduced risk of preterm birth, and improved experiences with care [7], [8]. It also promotes minimal use of interventions [7], [9], [10], including reduced rates of episiotomy, epidural and instrumental births, and efficient use of finite healthcare resources [8].

Midwife-led birth centres (MLBCs) provide an ideal environment for midwives to provide midwife-led care with autonomy [11]. An MLBC can be defined as *“a dedicated space offering childbirth care, in which midwives take primary professional responsibility for birthing care”* [12]*.* The scope of services in the MLBCs may extend beyond childbirth to include other services such as family planning, reproductive health, antenatal and postnatal care [12]. The choice of women to give birth in the MLBC is often complex, driven by availability of a multidisciplinary team and the desire for a positive childbirth experience typified by minimal use of childbirth interventions [10].

Most of the research evidence on MLBCs is from high-income settings, but there is evidence of the existence of MLBCs in many low and middle-income (LMICs), including Uganda [12]. Previous studies from LMICs, mostly countries in Asia, have attempted to explore perceptions of women regarding care offered in MLBCs [12], [13]. This study was part of a larger MLBC project conducted in Uganda, Bangladesh, Pakistan, and South Africa to explore the enablers and challenges for operating MLBCs in LMICs [14]. The aim of this paper is to explore the perceptions of women and midwives regarding the quality of care provided in MLBCs in Uganda, and identify reasons why women chose to give birth in MLBCs rather than other options.

## Methods

### Study design and setting

This was a descriptive qualitative study using an appreciative enquiry technique [15]. Appreciative enquiry focuses on identification of positive successes, but also enables participants to suggest solutions to the existing problems [15]. A Networks of Care (NOC) framework guided the design and implementation of the study [16], [17]. The NOC is a comprehensive framework for conceptualising enablers and challenges in a healthcare system such as in MLBC [16], [17]. MLBCs in Uganda are legally acceptable places of health care and are identified as maternity homes [18]. Midwives are legally permitted to own and operate MLBCs [18]. The MLBCs in Uganda are mostly situated in rural settings, standalone, privately owned units with variable funding mechanisms.

The study was conducted in four privately-owned MLBCs which were purposively selected from three diverse regions. One of the MLBC was constructed to resemble the rounded-shaped traditional homes in the local area.

### Study population and sampling procedure

We invited eight women who had used MLBC services, and 16 midwives working in MLBCs to participate in the study. Purposive sampling was used to select two women per site to participate in the study, while convenience sampling was used to invite all midwives working in the MLBCs. The number of women to be selected per site was pre-determined in the beginning of the study.

### Data collection procedure

Data were collected by focus group discussions and individual interviews. There were three focus group discussions with midwives, two individual interviews with midwife proprietors, and eight interviews with women who were the users of the services in MLBCs. Data collection was conducted between November 2022 and January 2023. An interview guide was developed to explore the four domains in the NOC: agreement and enabling environment, operational standards, quality efficiency and responsibility, and learning and adaptation [16]. The questions covered why women chose to give birth in the MLBC (Appendix 1). Five researchers (all nurse-midwives, one male and 4 female) and two research assistants (both nurse-midwives, one male and one female) conducted the data collection. The interviews and focus group discussions were audio recorded, transcribed and where necessary translated into English for analysis.

### Data analysis and rigour of the study

Data analysis was conducted by the team in Uganda. Thematic analysis was used to analyse the data [19]. We used both inductive and deductive approaches to map out the themes in the data. The inductive approach enabled identification of codes, sub-themes and themes were from the data, while in the deductive approach themes derived from the data were then mapped to frameworks of respectful maternity care [20], woman-centred care [21], and WHO quality of care [22]. Data analysis for the data from women were conducted first followed by data from the midwives. Subsequently, the analysis for both women and midwives were combined in the results section.

The credibility of the study findings was maintained through prolonged engagement with the participants during the interview, having the research team who led the larger four-country project review the findings and the use of nurse-midwives who were familiar with the Ugandan context and health system to conduct interviews [23]. Triangulation of data collection methods, sources of data (midwives, women), and study sites further ensure the rigour of the study findings.

## Results

Three themes were derived from the data: 1) Providing respectful, and dignified care 2) a focus on woman-centred care, and 3) choosing care in the MLBC (Table 1).

**Table 1:** Themes and sub-themes.

Theme	Sub-theme
Providing respectful, and dignified care	Receiving and providing non-discriminatory care Having care that is dignified Having companionship and not being abandoned Ensuring privacy and consent
A focus on woman-centred care	Receiving and providing holistic and individualised care Enabling empowerment and autonomy Having responsive providers Experiencing continuity of care Promoting confidence in the woman’s abilities Facilitating a positive birth experience
Choosing care in the MLBC	Care that is affordable Care that is available and accessible Care that is timely and responsive Care that is effective and comprehensive Having an effective referral system

### Providing respectful and dignified care

MLBCs provide care based on the midwifery model of care, a central tenet of which is respectful and dignified care. Women who gave birth in the MLBC were impressed with this aspect of the care they received. They contrasted the respectful care they experienced while in the MLBC with the disrespectful and abusive treatment they had previously experienced from the mainstream healthcare system. Their experience cemented the women’s resolution to seek future care in the MBLC.

#### Receiving and providing non-discriminatory care

Women reported receiving equitable service in the MLBC – they perceived that women using the MLBC were treated the same regardless of characteristics such as ethnicity, social status, or religion. This made them feel that they were as valued as the other MLBC clients. One woman said:

*“…They [the midwives] work with you just the way you are. They don’t segregate that this one is wealthy. I need to care for her more; she might give me money. No. Whether you’re poor, whether you’re wealthy…or you are like in which condition……they welcome you….”* (Woman, MBLC 3)

Midwives equally observed that the non-discriminatory care encouraged women to come to the MLBC explaining:

*“….for us here, we don’t segregate. We handle [treat] every mother…”* (Midwife, MLBC 3).

#### Having dignified care

A desire for dignified care was one of the most common reasons given for seeking care in the MLBC. Women described dignified care as being treated well, humanely, and with respect. The women contrasted this with the non-dignified care commonly found in the mainstream healthcare system, which they described in terms of mistreatment, humiliation, or abuse, for example:

*“Yes. It was there [respect]. You will also feel that I am treated as a human being”* (Woman, MLBC 2).

*“If you go to a place, maybe this side you’re abused, the other side you’re not abused. Where would you prefer? You will prefer where you will not be given ulcers.”* (Woman, MLBC 2).

Midwives similarly confirmed that women were treated with respect. This was thought to make women chose to give birth in the MLBC because of the respectful and empathetic care provided there, saying:

*“I don’t bark at them. I welcome them…. We become friendly when we are talking.”* (Midwife and MLBC proprietor, MLBC 4).

*“….I think one of the things that makes them trust this birth centre…when you come, you are treated with respect, you are listened to, you are treated with care and love. “* (Midwife, MLBC 3).

#### Having companionship and not being abandoned

Women appreciated that they were not left alone during labour and childbirth. The midwives were always there for them. This was very important, especially for those who came to the facility without a companion to attend to their non-medical needs. Midwives from the MLBC accompanied women who were referred from the MLBC to another facility and continued to care for them until they were discharged from the referral facility. Women explained:

*“… Because of labour pains then the nurse*^1^*said let me walk with you…She was the one who was there to help me until when I gave birth.”* (Woman, MLBC 2).

“*Like giving her all the time, asking all the questions. Appreciating her weaknesses and not to blame her for anything.*” (Midwife, MLBC 2).

#### Ensuring privacy and consent

Women and midwives noted the privacy and consented care offered in the MLBC. Women were allocated a private room where they could give birth. This was unlike the mainstream maternity hospitals where women give birth in an open room/ward sometimes with no curtains to separate the beds. The few private rooms available in the hospital maternity units are often reserved for those who could pay for them. A midwife explained how the service worked:

*“…They [MLBCs] offer more privacy, yes, more than some other [obstetric-led] facilities. Because sometimes other facilities, if you need a [private] room, you’ll have to cough some money….but here you are offered your room with no cost as long as you are a client.”* (Midwife, MLBC 3).

Women highlighted the value of privacy as explained here:

*“I was put in my separate room and closed. It was not open for anyone to see what I am. They kept my dignity.”* (Woman, MBLC 4).

*“Maternity homes [MLBCs] are different from other facilities, for example, you sleep in single rooms for one person…”.*(Woman, MLBC 3).

*“They would involve you. They will tell you we are going to examine you so get on the examination bed, they tell you.”* (Woman, MBLC 4).

### A focus on woman-centred care

Woman-centred care involves prioritising each women’s unique needs, providing culturally sensitive care, empowerment and autonomy in decision-making, continuity of care, creating a safe space home-like environment for women, and responsive care providers [24].

#### Receiving and providing holistic and individualised care

Women described how the care in the MLBCs was tailored to meet their individual needs, including physical (e.g., labour pain relief), social (e.g., privacy), emotional and cultural needs (e.g., communication in her mother tongue, made possible because the midwives were mostly from the local community). Cultural sensitivity was supported by the design of the MLBC, which mimicked the home environment. Women gave examples here:

*“They listen and indeed, I experienced this care during labour when they rubbed my back when I was in pain during labour, they comfort you.”* (Woman, MLBC 3).

*“They allowed my husband to come to the labour ward…. the staff used my local language during communication. So, language was not a problem.”* (Woman, MLBC 3).

*“… If you come alone, they will be asking you that is there no one who has accompanied you?….they will ask how you are feeling. You might say that hunger is a problem, and they may get you tea.”* (Woman, MLBC 2).

Midwives in the MLBC were able to meet their client’s individual and holistic needs through a deliberate effort to understand and listen to the women. They explained their approach as:

*“…We understand the complete situation of each client that we have….we understand if that client has a husband who beats her, we understand what is going on in her home. If she is one of many wives, we understand….we very closely understand not only the situation but also the needs of our client because those things impact maternal health…so, understanding the whole picture means that our clients also trust us more…”*(Midwife, MLBC 3).

*“…We make sure that the women are heard. We make sure we also understand their needs, then we make sure we are able to provide their needs, the needs of the clients.”* (Midwife, MLBC 3).

One of the MLBCs made good use of traditional birth attendants (TBAs). Although TBAs are not officially recognised as part of the Ugandan health workforce, they are significant and trusted community members. They serve as mobilisers to promote and encourage women to come and give birth in the MBLC, but also their presence in the MLBC served to cement the trust the community had for the MLBC. The midwife explained:

*“…so putting them [TBAs] at the centre, we created a birth centre that is really for the community. They have their trusted people who are usually the TBAs and then the TBAs trust us. So, they bring them to the facility and we offer a small pay so they get compensated for every person that they bring to the facility.”* (Midwife, MLBC 3).

#### Enabling empowerment and autonomy

Although women were used to accepting health care passively without questioning, and lacked the agency to demand participation in decision-making, the MLBC midwives encouraged them to ask questions regarding their care. Midwives then supported their decisions and advocated for them, especially if they were transferred to referral sites. The women felt at ease with the midwives and, as such, felt they had the freedom to ask questions at will. The midwives provided information to the women which was reassuring and empowering for them. For instance:

*“…They allow us to ask questions because they tell you if you have anything to ask us, please do ….So you are given a chance to ask whatever you want to ask.”* (Woman, MLBC 1).

*“Yes. you can ask. Like now, if I come and there is something I don’t understand, I can also ask. They can even sometimes tell you that don’t fear, you ask which means I will also ask…they allow me to ask about everything*.*”* (Woman, MLBC 2).

#### Having responsive providers

Women in the MLBCs enjoyed a positive relationship with the midwives. The women often described the midwives as *‘like sisters or mothers’* indicating a familial-like relationship. The positive relationship was responsive and devoid of arrogance, pride, and resentment towards women, which they said was often seen in the mainstream healthcare facilities*.* The midwives worked with a smile and warmly welcomed the women to the MLBC, which encouraged the women to confide in the midwives freely. These women explained:

*“They are not arrogant….and their [good] manners, they are not people who discriminate, they are not ill-mannered. They received me [well]….she managed to be there for me like my sister.”*(Woman, MLBC 2).

*“…They are ever happy … all of them have smiles and even when giving you results, they are always jolly and you feel you can confide in them … when you see a person with a smile, you smile too and you talk as if you are home and you may forget that you are in the health facility.”*(Woman MLBC 3).

Midwives working in the MLBC underwent some additional training as part of their induction into the MLBC workforce. The training enabled them to build effective relationships with the women during the course of care. They explained that:

*“…Once we get a midwife, we first train all our midwives…we have our tradition of how we treat our mothers. So, after the training…they know how to build relationships with clients. Sometimes, if you find a midwife with a client, you may not even differentiate who is a midwife and who is a client because the bond is very strong.”* (Midwife, MLBC 3).

The women especially those who were young trusted the midwives working in the MLBC as the experts of pregnancy and childbirth.

*“We trust everything that she does because she is very experienced….So, if I come and she tells me to do something, I agree because I know she will help me to recover.”* (Woman, MLBC 4).

#### Experiencing continuity of care

The MLBC often had the same midwives providing care across the maternal and child health continuum. The integrated services in the MLBC made continuity of care possible which enabled women to be served without delay by familiar providers, for example:

*“Because that day when I came, … they straightway received me, and they knew me because I used to come here for antenatal care….”(Woman, MLBC 2).*

*“We also give continuous care. And let’s say, we have mothers who have had their fifth babies with us, so when they are coming back, they know exactly what to expect and what not to expect. So, coming back they find it even if it’s a new face of a midwife but they still find like a photocopy of the other one: with a good heart, loving, and caring and supporting them.”* (Midwife, MLBC 3)

#### Promoting confidence in the woman’s abilities

Care in the MLBCs involved supporting the physiological process of childbirth. The midwives supported, encouraged, and reassured the women during the labour process. The support was in the form of providing enough information, leading, morale boosting, and reassurance in the face of fear. This made the women feel safe and empowered. Women said:

*“I pushed but I did not have enough energy by then. So, she kept on encouraging me to push that she was also assisting me.”* (Woman, MLBC 2).

*“I got shocked because I was told that if you reach there [referral hospital], you will be operated. That made me fear…, I asked the midwife will I be operated? She said only that you are going to be strong… You are going to give birth.”* (Woman, MLBC 2).

A midwife also explained:

*“So, we ensure we don’t force anything…we prefer our client to follow her body instructions. Then we, we believe in her body instructions. Sometimes, it brings out magical results [laughs].”* (Midwife, MLBC 3).

#### Facilitating a positive birth experience

Midwives endeavoured to facilitate a positive birth experience for women by providing a home-like environment that made women feel comfortable and safe. In one MLBC the labour suite bed was made to resemble traditional beds commonly found in the local homesteads in the area and was low to help the woman feel safe. Other examples were:

*“So, we make sure those moments [childbirth], we create a loving memory for her to take with her; not to forget, not a memory that will traumatise, confuse them or depress them*.” (Midwife, MLBC 3).

*“Like I have not seen these structures anywhere. Or else, I have not seen any labour suite bed this low…., here we do cherish, we listen to the woman’s body… So, with the low bed, it encourages and supports the woman to feel safe and change position without worrying about falling off the bed. So, I think that was the whole idea of having it low and comfortable as a patient would have it at home.”* (Midwife, MLBC 3).

*“…when you are in the community, you’re a community birth centre and everyone comes to visit all the time. You know, it’s not like in hospital, [where] you deliver and never come back to visit. Everyone considers this their home in many ways….”.* (Midwife, MLBC 3).

Midwives in the MLBCs prioritised creating a peaceful environment for women. This was through the midwifery model of care. This model of care was perceived to be empowering for both the women and the midwives. They said:

*“We also believe in creating a peaceful environment for the women as treatment. So, meanwhile, elsewhere you’d go then at times they believe in drugs only but sometimes not realising that space, a peaceful space, being listened to is also treatment…”* (Midwife, MLBC 3).

### Choosing care in the MLBC

The services in the MLBCs were perceived in the eyes of women as available, accessible, effective, responsive, affordable, comprehensive, and integrated within the wider health system. Thus, they matched the needs and expectations of the women.

#### Care that is affordable

The services in the MLBCs which relied on user fees were deemed by women to be fairly affordable and facilitated by a flexible payment system. The flexibility was possible because the women were known to the care providers, which made it easier for them to obtain care first and pay later. In the MLBC that was financed by external funding, a small charge of 5000 Uganda shillings [1.5 USD] was levied, served as a token of appreciation for the TBAs associated with the MLBC. External donations and governmental support met other costs. One midwife said:

*“They [women] are never overwhelmed with the costs because they feel more of the service here is more of free….as compared to government facilities….like, you see after delivery here, they get some pieces of clothes. Then they pay 5000 shillings…so, I think even that support is the one which encourages them…to come back here…”*(Midwife, MLBC 3).

Besides the affordable care, women were given supporting materials like baby clothes after delivery which they greatly valued. One said:

*“They [medical bills] are not as high like in other hospitals…my husband may first ask me to come…he may first say go there for treatment; they won’t refuse because they know you. He will come later and they will tell him the medical bill [to pay].”* (Woman, MLBC 2).

#### Care that is available and accessible

In Uganda, most MLBCs are located in underserved rural areas with poor road networks and distant from hospital settings. Women in these locations preferred to go to the MLBC because it was nearby, accessible, and convenient. Unlike in the mainstream health facilities where the personnel, drugs, and medical supplies may be unavailable, women preferred MLBCs because all services were available to them, for example:

*“They are there because for me usually when I come, I find the items there. Those things of testing, if you’re pregnant those things of listening for the fetal heartbeat…. Because when I come, I find when they are there.”* (Woman, MLBC 3).

*“Compared to the government facility where sometimes you go, you bring your mother, you find there’s no midwife. You go there to knock, knock, there is nobody answering you. But here they are just 24 hours waiting for you day and night in case of anything.”* (Midwife, MLBC 3).

#### Care that is timely and responsive

Women in the MLBCs were impressed with the short waiting time, timely care, and immediate response to their needs. Women valued the short waiting time as it enabled them to return home in time to complete their household chores. Women noted that they were given more contact time with the healthcare provider. They explained:

*“It is so good because when you come here, they work on you quickly and you go back home. You do not come and have to wait for long to be attended to. Also, they give you time.”* (Woman, MLBC 1).

“*What makes them special is that they do their things on time, they do not make you wait like other hospitals … And you are given more time than the other side because the other side you can call the midwife and she does not care … which is not here … the midwives here treat you well as if you are her sister or mother.*” (Woman, MLBC 2).

#### Care that is effective and comprehensive

Effective care services were a recurring theme for choosing to come to the MLBCs. Effective care was described by women in terms of being comprehensive, which enabled them to fully recover from pregnancy and labour. For instance:

*“….and their treatment is good. If you’re sick, you are treated well…”* (Woman, MLBC 2).

*“….I get all my healthcare from here. Everything I get from here….”* (Woman, MLBC 2).

Midwives observed that effective care in the MLBC translated to having positive birth outcomes. This was through adopting evidence-based practices like delayed cord clamping, exercises in labour, and promoting physiological processes of childbirth. Two midwives explained:

*“…you know, one of the things we have been able to address is to ensure that every mother and the baby walks out of the facility healthy, thriving, and alive…”*(Midwife, MLBC 3).

*“.It’s a special birth center and we are really trying to show that we can do something different and have really good outcomes….that having good outcomes is not about having big fancy machines, about being in the biggest hospital, it’s about having real midwifery models of care. So, like real like midwifery care that is aligned to the midwifery model of care and I think that’s what is missing in most health centers in Uganda.”* (Midwife and MLBC proprietor, MLBC 3).

#### Having an effective referral system

Some of the MLBCs established an effective referral system where women were identified and picked up from their homes then taken to the facility, and also transported from the MLBCs to a referral site in the event of complications. The MLBCs had transport in the form of an ambulance, tricycle, and motorcycle which were deemed acceptable to the women. The midwives often escorted the women during the referral process, which was reassuring for the women. The MLBCs established a memorandum of understanding with the referral site, including two-way communication. This worked well as explained here:

*“From home to [MLBC] I was picked up by the ambulance sent by [MLBC]. I just made a phone call. The telephone number was given to me during antenatal. When you make a phone call, they pick you [up]. My journey was easy, this should continue [laughs].”* (Woman, MLBC 3).

*“We handle them very well. If there’s something which we know that maybe for us we shall not complete from here, we make a referral….the mother should also feel that, ehh, these people have really helped me and they transfer me. Now my baby is now very ok.”* (Midwife, MLBC 3).

## Discussion

The study explored women’s perceptions regarding the quality of care in the MLBCs. Overall, the women perceived that care in the MLBC to be respectful and dignified. Women described the environment and relationships in the MBLC in terms of home-like settings, which made them feel comfortable, and it provided a platform for respect of autonomy and involvement of women in decision-making about their care. The care in the MLBC was rated positively in relation to availability, accessibility, and affordability of services as well as effective care and referral system, which were congruent with the expectations of women.

Previous studies have underscored the widespread disrespect and abuse of women in hospital settings and the corollary preference of women to give birth in a home-like environment without risk of obstetric violence [24], [25], [26], [27], [28]. Women in our study preferred to give birth in the MLBC because of the respectful and dignified care. The midwifery philosophy of care implemented in the MLBCs has respectful care at its core, underpinning on the philosophy of respect for human dignity, justice, equity, and partnership with women [7], [9]. The respectful care in the MLBC was quite surprising to women, probably because of the normalisation of mistreatment in the obstetric-led units [25]. Consequently, women compared the respectful care they received in the MLBCs favourably with their previous experience of mistreatment in the obstetric-led units. The respectful care in the MLBC was consistent with the preference of women to use home-like metaphors cited in the literature to describe peaceful experiences in MLBC [5]. Respectful care in the MLBC strengthened the resolution of women to seek care in the MBLCs for future pregnancies. Mistreatment of women demands the development of models of care that encourage respectful care during childbirth [25], [26].

Midwife-led care, especially in an MLBC, values creating a meaningful birth experience through women-centred care that meets the woman’s individual needs [8], [24]. Although women in our study seemed to lack agency and empowerment in their day-to-day lives, midwives supported and empowered them by encouraging questions and involving them in decision-making. This may have knock-on effects on women’s empowerment more generally and thus contribute towards SDG 5 [29].

The responsive providers made women describe midwives using familial-like metaphors, which suggested a lack of formalities, social hierarchy, and bureaucracy that are often seen in hospital maternity units [5]. The organisational setup of services in some of the MLBCs in our study, including housing structures that mimicked the traditional homes in the locality, further cemented the home-like environment in some of the MLBCs. Woman-centred care occurred in this context of the familial-like relationship where midwives were able to identify with the women and consequently meet their individual needs [5], [24]. In our study, culturally appropriate interventions were used, particularly for pain relief, fears and capabilities of childbirth, which was consistent with the midwifery model of care [5], [10].

In high-income countries, women may choose to deliver in MLBCs because of their concern about avoiding over-medicalisation [10]. This concern was also evident in our study, but the women were even more concerned about getting *“good service”,* which they described in terms of convenience, availability, accessibility, hospitability, comprehensiveness, and affordability. In LMICs, MLBCs often act as government extensions of services to rural populations with poor access to essential services [30]. This helps to explain the high priority placed by the women in this study on accessibility of services. It also highlights a fundamental lack of alternative models of care available to women in these settings, which raises the question of whether women are opting for MLBC care only because they have little choice or a lack of individual agency, empowerment, or autonomy. Our findings suggest that this is not the case – women valued MLBC care because of its perceived benefits and strengths. This is important to note within a wider societal culture of medical dominance and overshadowing of the midwifery model of care [5], [26].

## Study strengths and limitations

The study describes in depth the perceptions of women and midwives from four different settings across Uganda, strengthening the study findings’ transferability. We did not collect data on the socio-demographic profile of the participants which may limit contextual understanding of the characteristics of the participants in the study. The study findings regarding the perception of care in the MLBC may be limited to only the perspective of the women and midwives. Further studies may explore the perception of male partners, family, the community, and other care providers, regarding quality of care in MLBCs.

## Conclusion

Women and midwives positively rated the overall quality of care across the four MLBCs. This was mostly because of respectful and dignified care, which included dignified care, non-discriminatory care, privacy, and consented care. Scaling up MLBCs has the potential to empower midwives and reduce the mistreatment of women during childbirth. The care in the MLBCs was perceived as women-centred, typified by meeting the women’s holistic individual needs, empowerment and autonomy, positive healthcare provider relationship, continuity of care, and confidence in women’s abilities. Women liked the care in the MLBCs because the services were convenient, available, accessible, affordable, and comprehensive. Future studies need to explore how to develop sustainable financing mechanisms to support the establishment and running of MLBCs.

## Ethics approval and consent of the participants

The study obtained ethical approval from the Mulago National Referral Ethics and Research Committee (REC- number: MHREC-2022–77) dated 9th September 2022 and administrative clearance from the Uganda National Council of Science and Technology (HS2795ES). The participants provided written informed consent.

## Funding

The study was funded by the Bill and Melinda Gates Foundation through a grant to the International Confederation of Midwives (ICM). The funders did not influence the study, and as a result, the content is solely the responsibility of the authors.

## Author contributions

RCN, AN, OB, KH, SCL, ST and CH participated in the conceptualization of the research idea and development of the study protocol. RCN, JE, FN, and SNM participated in obtaining the ethical clearance for the study. RCN, JE, FN, SNM, and SN conducted data collection and analysis. RCN wrote the first draft of the manuscript, while JE, FN, SNM, AN, SN, AN, OB, KH, SEL, ST, MF, and CH revised the first draft of the manuscript. All the authors met the criteria of authorship and approved the final draft for publication.

## Declaration of Competing Interest

The authors declare no competing interests. Caroline Homer is the Editor-in-Chief of Women and Birth and as such played no role in the decisions around this paper. The Deputy Editor, Linda Sweet, managed all decisions and correspondence.

## Data Availability

The additional data and materials can be accessed from the corresponding author on a reasonable request.

## Footnotes

1 Women tended to use the word ‘nurse’ when referring to the nurse-midwives who led their care.
