# Supplementary material for: Perceptions of quality of care in Midwife-led Birth Centres (MLBCs) in Uganda: Why do women choose MLBCs over other options?
Source: Women Birth. 2024 Jul;37(4):None. doi: 10.1016/j.wombi.2024.101612 (PMC11266915; doi:10.1016/j.wombi.2024.101612)
Supplement: Supplementary file 1 — Supplementary material. [file mmc1.pdf]

Guiding questions in Focus group discussions with Health workers
Introductory questions:

- Explore the respondent's role in the MLBC (ask for their gender, how long they have worked there, was it their own choice to work there or were they just deployed there, what is their role in MBLC?)
- Please describe your colleagues at the MLBC (ask for who else works with you, how do their roles and responsibilities compare to yours, who is in charge (and what does "being in charge" look like)?

Key questions for focus group discussion:

- What type of support does government provide for Maternity centers? (probe for financial, supplies, mentorship/training/supervision, guidelines)
- How do you engage or involve the community? (How do you get buy-in from community?)
- What does an ideal relationship between an MLBC midwife and her client look like
- As a midwifery provider, tell us what you do to make women trust the MLBC services (probe why)
- How do you address the specific needs of the community? (probes: Are there any barriers or enablers? What would you do to make the services more acceptable by the community?)
- What is the main payment method for service users? Which women can afford MLBC services? Does the government or other organisations arrange any financial support for the service users? (Explore in detail)
- How do you ensure that the MLBC has all the supplies, equipment and resources it needs to provide high quality services?
- What kind of information do you record about MLBC performance? (probe: what data management system do you have or how do you keep records, how is it linked/integrated to the district/national data base)
- How do you handle emergency/ complicated cases in the MLBC? (Probe: Where do you refer the client? What kind of agreement do you with other referral health facilities? How do they get there?) What does it look like when the referral system works well?
- What are the best things/moments for you as a provider working in MLBC? (What do you enjoy the most?)
- How do you feel about working within MLBC?
- What would you like to change in future to make the situation better for you? (Probe for burnout, supports and workload)
- What competencies do providers need to work in an MLBC? Do you think all health care providers in this MLBC have all the required competencies? If not, what should be done to improve it?
- How do you ensure that the care that you provide is evidence-based? What main guidelines and standards do you use? Are those guidelines helpful? What else needs to be done?

- If you were planning to improve the quality and efficiency of MLBC services, what would be the three main things you would suggest?
- In what ways is the care provided client/women-centred?
- Is there something innovative and unique that your MLBC provides and that facilities do not? (Apart from regular services you are providing) How does this benefit the clients and/or the health care providers?
- Is there any modern approach or technology that you are using as part of MLBC services? How does this benefit the clients and/or the health care providers? What kind of technology or approaches would improve services in future?
- How do you facilitate access to MLBC services for those who might find it more difficult to access care here, e.g. because they are poor or cannot easily travel? How could you improve this in future?

Guiding questions in Key Informant Interviews with women
Introductory questions:

- Tell me about your most recent birth at (name of MLBC).
- When was it? Did you have a son or a daughter?
- Was it your first birth? If not, where did you give birth before?

Key interview questions

- How did you know about this maternity center and why did you choose it?
- What did you like about the maternity center services?
- What did you like about the staff of the Maternity center? (Feel comfortable to share things or ask questions)
- How did the maternity staff involve you and your family in decision making about your care?
- In what ways did the MLBC respect your needs? (Probe for things like: birth partners, language, respect for cultural traditions that are important to the woman)
- What or who helped you to pay the costs of accessing care? (Probe as appropriate for: user fees, transport costs, food and accommodation for self and family members, medicine costs, equipment costs (e.g., sanitary pads)
- Would you recommend the MLBC services to other women? If yes or no why?
- What are the three main things to be changed for better services in future?
- Do you think the MLBC has enough health workers, materials and equipment to provide high quality childbirth services? (probe if yes or no why). What should be done to make it better in future?
- What did the midwives do to make you feel confident that they knew how to do their job well?
- What did the midwives do to make you feel confident in your own ability to give birth safely and care for your baby?
- What documentation and paperwork did they give you when you were discharged from the MLBC?
- Before you gave birth, what information did the MLBC staff give you about what would happen if there was a complication or emergency that meant you needed to transfer to a hospital?
- Did you or your baby need to be transferred to another facility either during labour or shortly after the birth? Why? Tell me about that experience. How did you feel?
- How did you make the journey from your home to the MLBC? What would have made your journey easier for you?
- Would you give birth at MLBC again in future, or recommend the MLBC to a friend or relative? Why?
- What are the things that could have been improved further? Please describe three main things you would suggest for improvement.
- What is it about the MLBC that makes it different from other health facilities where women can give birth?
- How did the midwives make you feel respected?
- How did the midwives encourage you to ask questions and ask for what you needed?
- How did the midwives encourage you to make your own decisions about your care?
